# In vitro susceptibility of nontuberculous mycobacteria in China

**DOI:** 10.1186/s12879-024-09016-6

**Published:** 2024-01-23

**Authors:** Weicong Ren, Yaoju Tan, Zichun Ma, Yuanyuan Shang, Shanshan Li, Xuxia Zhang, Wei Wang, Cong Yao, Jinfeng Yuan, Liang Li, Yu Pang

**Affiliations:** 1 Department of Bacteriology and Immunology, Beijing Key Laboratory on Drug-Resistant Tuberculosis Research, Beijing Tuberculosis & Thoracic Tumor Research Institute, Capital Medical University, Beijing, China; 2 Department of Clinical Laboratory, Guangzhou Chest Hospital, Guangzhou, China

**Keywords:** Nontuberculous mycobacteria, Drug resistance, China

## Abstract

**Objectives:**

This study aimed to measure the prevalence of resistance to antimicrobial agents, and explore the risk factors associated with drug resistance by using nontuberculous Mycobacteria (NTM) isolates from China.

**Methods:**

A total of 335 NTM isolates were included in our analysis. Broth dilution method was used to determine in vitro drug susceptibility of NTM isolates.

**Results:**

Clarithromycin (CLA) was the most potent drug for *Mycobacterium intracellulare* (MI). The resistance rate of 244 MI isolates to CLA was 21%, yielding a minimum inhibitory concentrations (MIC)_50_ and MIC_90_ of 8 and 64 mg/L, respectively. 51% of 244 MI isolates exhibited resistance to amikacin (AMK). For 91 *Mycobacterium abscessus* complex (MABC) isolates, 6 (7%) and 49 (54%) isolates were categorized as resistant to CLA at day 3 and 14, respectively. The resistance rate to CLA for *Mycobacterium abscessus* subspecies *abscessus* (MAA) was dramatically higher than that for *Mycobacterium abscessus* subspecies *massiliense* (MAM). Additionally, the percentage of patients presenting fever in the CLA-susceptible group was significantly higher than that in the CLA-resistant group.

**Conclusions:**

Our data demonstrate that approximate one fifth of MI isolates are resistant to CLA. We have identified a higher proportion of CLA-resistant MAA isolates than MAM. The patients caused by CLA-resistant MI are at low risk for presenting with fever relative to CLA-susceptible group.

**Supplementary Information:**

The online version contains supplementary material available at 10.1186/s12879-024-09016-6.

## Introduction

Despite being considered opportunistic pathogens, infections due to nontuberculous *mycobacteria* (NTM) are significantly increasing worldwide becoming a major public health threat [1, 2]. These bacteria are able to manifest a large spectrum of diseases. Pulmonary infections account for approximately 90% of all NTM-associated diseases; the rest involve lymph nodes, skin and soft tissue, and bones [3, 4]. The clinical course and treatment response differ depending on the causative NTM species, which vary in their susceptibilities to antibiotics of potential therapeutic use [5, 6]. Therefore, precise species or subspecies identification of NTM clinical isolates is of great importance for managing patients with NTM diseases [7].

Compared with tuberculosis (TB), the epidemiologic data of NTM infections are notifiable in relatively few regions. Limited evidence generally shows that the incidence of NTM infections has dramatically increased worldwide [8]. In clinical practice, patients with NTM diseases are considered as candidates for antibiotic multidrug therapy [7]. The drug susceptibility testing (DST)-guided therapy will thus provide benefits in improving treatment outcomes of NTM patients. Unfortunately, treatment recommendations by the American Thoracic Society and Infectious Diseases Society of America (ATS/IDSA) criteria are majorly based on expert opinions and traditions [9]. The geographic diversity in drug resistance of NTM isolates impairs the clinical efficacy of experience-based regimens [10–12]. As a consequence, regional surveys for antibiotic resistance are essential for formulating optimal recommendations for the management of NTM diseases. The available evidence base was not derived from representative samples or standardized laboratory results. These limitations prevented an adequate assessment of the extent of the problem.

China has the third largest tuberculosis (TB) epidemic globally [13]. Despite great achievements in TB control over past decades, population-based data demonstrates a significant increase in NTM incidence rate in this country [14], attracting attention from a public health perspective. In 2019, the China *Nontuberculous Mycobacteria* Surveillance Study (CNTMS) was initiated to explore the proportion of NTM disease among patients with symptoms suggestive of pulmonary TB [2]. By using a representative set of NTM isolates, we aimed to measure the prevalence of resistance to antimicrobial agents with the use of standardized methods, and also determine the risk factors associated with drug resistance.

## Materials and methods

### Bacterial isolates

All NTM isolates (*n* = 348) were collected from the China Nontuberculous Mycobacteria Surveillance Study (CNTMS), a nationwide cross-sectional study in China consecutively enrolling smear-positive patients with clinical symptoms suggestive of tuberculosis from December 2019 to June 2020 as previously reported [15]. All NTM isolates obtained from several hospitals in different regions of China were transferred to the Beijing Chest Hospital, Capital Medical University, for further analysis. Species identification was performed using the MeltPro Myco assay. For the *Mycobacterium abscesses* complex (MABC), sequencing of *rpoB* and *hsp65* were done using previously recommended criteria [14]. The positive cultures were collected in 7H9 medium supplemented with 10% of oleic acid-albumin-dextrose-catalase (OADC) and 5% glycerol, and stored at -70℃. Prior to in vitro drug susceptibility testing, the isolates were subcultured on the Löwenstein-Jensen (L-J) medium at 37 °C for one week or four weeks depending on growth rate of *mycobacterial* species. Multiple demographic and clinical characteristics were obtained from the electronic patient records in each hospital. This study was approved the Ethics Committee of Guangzhou Chest Hospital for the anonymous use of *mycobacterial* isolates with a waiver of informed consent.

### Phenotypic drug susceptibility testing

The minimum inhibitory concentrations (MICs) of antimicrobial agents against clinical NTM isolated were measured using broth dilution method in cation-adjusted Mueller-Hinton broth recommended by the Clinical Laboratory Standards Institute (CLSI) [16]. The commercially available plates for slowly glowing mycobacteria (SGM, Sensititre Myco SLOMYCOI assay) and rapidly growing mycobacteria (RGM, Sensititre Myco RAPMYCO assay) were purchased from Thermo Fisher (Waltham, USA). Briefly, we scraped the fresh colonies from the L-J slant. A suspension of NTM isolate was adjusted to the density of a 0.5 McFarland standard. After dilution 1:200 in cation-adjusted Mueller-Hinton broth, and brought on Sensititre plates at 100 µl per well. Plates were sealed and incubated at 37 °C for SGM and 30 °C for RGM. MICs for the RGM were read after incubation for 3 to 5 days when sufficient growth was observed in the control well. The SGM were read after incubation for 7 to 14 days until sufficient growth was observed in the control wells. To determine the inducible clarithromycin (CLA) resistance of MABC isolates, the plates were further incubated for 14 days at 30 °C for a final reading. The growth of mycobacteria was monitored by the Sensititre Vizion system with data analysis using the SWIN software package (Thermo Fisher Scientific). Quality controls *Mycobacterium marinum* (ATCC700686) and *Mycobaterium peregrinum* (ATCC14467) were used as positive control per batch experiment for SGM and RGM, respectively. The detection range of MIC for quality control of these two control strains was referred to table S1 and table S2 in CLSI document M62 [16].

### Interpretation of susceptibility results

MIC_50_ and MIC_90_ was defined as the lowest concentration of the antibiotic at which 50% and 90% of the isolates were inhibited, respectively. The interpretation of MIC results followed the criteria as stated in CLSI document M62 [16]. Thirteen antibiotics were used: CLA, amikacin (AMK), moxifloxacin (MXF), linezolid (LZD), rifabutin (RFB), rifampin (RIF), ciprofloxacin (CIP), doxycycline (DOX), ethambutol (EMB), trimethoprim-sulfamethoxazole (TMP-SMX), cefoxitin (FOX), imipenem (IPM) and tobramycin (TOB). MIC breakpoints (susceptible, intermediate, and resistant) were interpreted in Supplementary Tables S1 and Table 2.

### Statistical analysis

The SPSS 20.0 (IBM Corp., Armonk, NY, USA) was used for the statistical calculations. Chi-square was conducted to compare the proportion of drug resistance between NTM species. Univariable analyses were performed to examine the association of each variable with drug resistance among NTM patients. The differences were considered to be statistically significant at *P* < 0.05.

## Results

### MIC distribution of antimicrobial agents against Mycobacterium intracellulare isolates

In our previous studies showed that the most prevalent species was *Mycobacterium intracellulare* (MI), followed by MABC in NTM pulmonary disease [15]. So we conducted drug susceptibility testing for MI isolates. After 254 MI isolates were successfully resuscitated, all of them were tested for antimycobacterial susceptibility testing, 10 isolates were failed and a total of 244 MI isolates were included in our analysis. The drug susceptibility profiles of MI, including MIC_50_ and MIC_90_, are summarized in Table 1. Overall, CLA was the most potent drug for MI. The resistance rate to CLA was 21% (52/244), yielding a MIC_50_ and MIC_90_ of 1 and 16 mg/L, respectively. The results also showed that 51% of MI isolates exhibited resistance to AMK (125/244), followed by LZD (169/244, 69%) and RFB (212/244, 87%). By contrast, high resistant rates were observed for MI isolates towards RIF, CIP, MXF, and trimethoprim-sulfamethoxazole, respectively.

**Table 1:** Antimycobacterial susceptibility testing results for clinical isolates of *Mycobacterium intracellulare*

Antimicrobial agents	Range (µg/ml)	MIC (µg/ml)		No. (%) of isolates		
		50%	90%	Susceptible	Intermediate	Resistant
Clarithromycin	0.06-64	8	> 64	143(59)	49(20)	52(21)
Rifabutin	0.25-8	8	> 8	63(26)	—	181(74)
Moxifloxacin	0.12-8	> 8	> 8	39(16)	7(3)	198(81)
Rifampicin	0.12-8	> 8	> 8	32(13)	—	212(87)
Trimethoprim-sulfamethoxazole	0.12/2.38-8/152	> 8/152	> 8/152	38(16)	—	206(84)
Amikacin	1–64	64	> 64	110(45)	9(4)	125(51)
Linezolid	1–64	64	> 64	62(26)	13(5)	169(69)
Ciprofloxacin	0.12-32	> 32	> 32	32(13)	4(2)	208(85)
Doxycycline	0.12-16	> 16	> 16	17(7)	2(1)	225(92)

MIC, minimum inhibitory concentration

### MIC distribution of antimicrobial agents against Mycobacterium abscessus complex isolates

We have found that the three most prevalent species were MI (52.6%), MABC (23.1%), Mycobacterium avium (8.5%) in NTM pulmonary disease [15]. Therefore, 94 MABC isolates were successfully resuscitated, all of them were tested for antimycobacterial susceptibility testing, 3 isolates were failed and 91 MABC isolates were studied, consisting of 69 *Mycobacterium abscessus* subspecies *abscessus* (MAA) and 22 *Mycobacterium abscessus* subspecies *massiliense* (MAM) isolates. Overall, 6 (7%) and 49 (54%) isolates were categorized as resistant to CLA at day 3 and 14 on the basis of CLSI breakpoint, respectively. AMK was also highly active against MABC isolates, the MIC_50_ and MIC_90_ of which were 16 and 64 µg/ml. Using 64 µg/ml as breakpoint, the resistance rate for AMK was 12% (11/91). FOX, LZD and TOB exhibited moderate activity against MABC isolates, and 29%, 49% and 40% of isolates tested were resistant to these drugs, respectively (Table 2).

**Table 2:** Antimycobacterial susceptibility testing results for clinical isolates of *Mycobacterium abscessus* complex

Antimicrobial agents	Range (µg/ml)	MIC (µg/ml)		No. (%) of isolates		
		50%	90%	Susceptible	Intermediate	Resistant
Clarithromycin (Day 3)	0.06-16	0.25	2	83(91)	2(2)	6(7)
Clarithromycin (Day 14)	0.06-16	8	> 16	42(46)	0(0)	49(54)
Amikacin	1–64	16	64	71(78)	9(10)	11(12)
Linezolid	1–32	16	> 32	39(43)	12(13)	40(44)
Moxifloxacin	0.25-8	8	> 8	4(4)	6(7)	81(89)
Ciprofloxacin	0.12-4	4	> 4	4(4)	8(9)	79(87)
Cefoxitin	4-128	32	> 128	22(24)	43(47)	26(29)
Imipenem	2–64	64	> 64	0(0)	14(15)	77(85)
Doxycycline	0.12-16	16	> 16	4(4)	6(7)	81(89)
Tobramycin	1–16	4	> 16	33(36)	18(20)	40(44)
Trimethoprim-sulfamethoxazole	0.25/4.75-8/152	> 8/152	> 8/152	0	—	91(100)

MIC, minimum inhibitory concentration

**Fig. 1:**
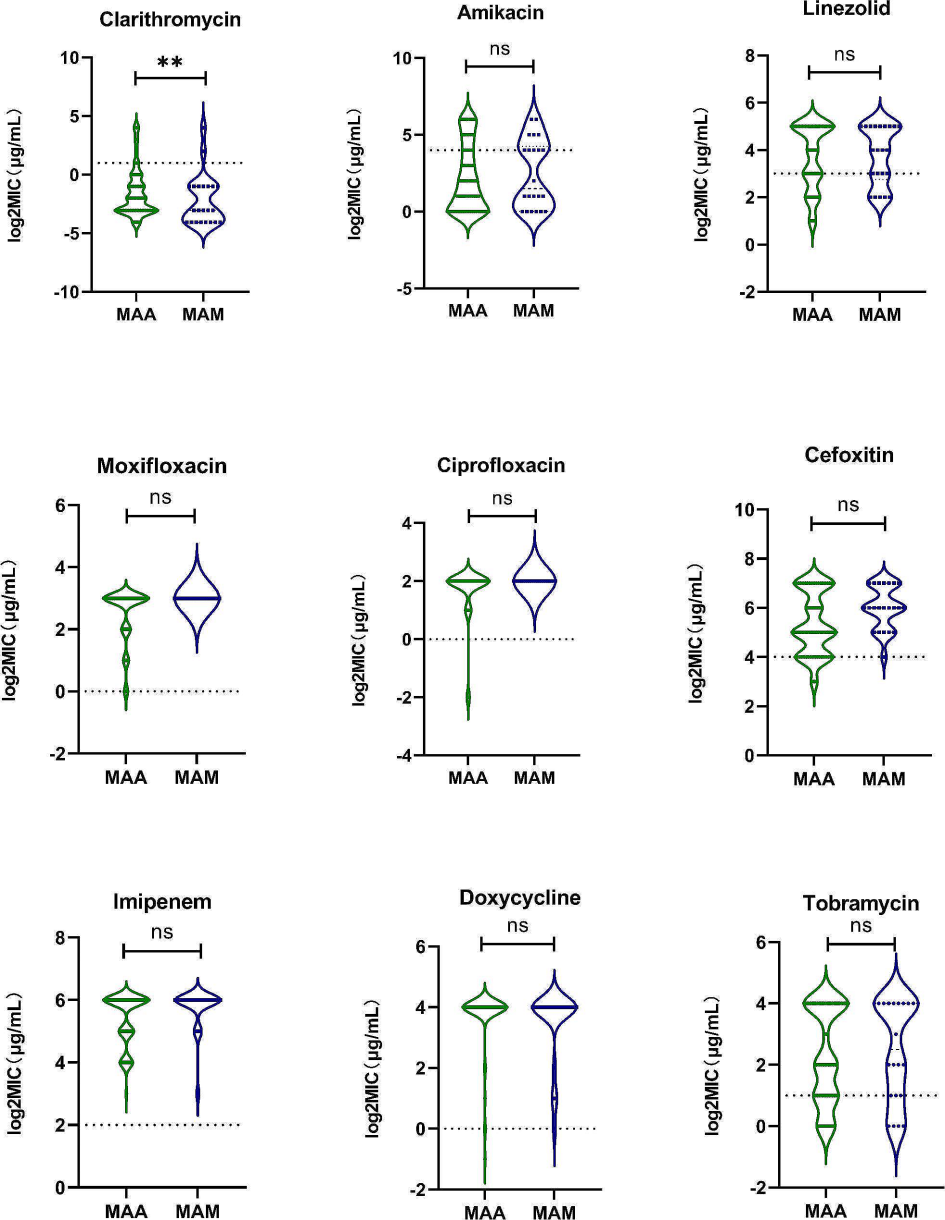
Comparison of MIC distribution between *Mycobacterium abscessus* and *Mycobacterium massiliense* isolates

We further compared in vitro susceptibility patterns between MAA and MAM. As illustrated in Fig. 1, higher MIC values were noted for MAA isolates toward CLA compared to MAM isolates. The resistance rate to CLA was 7% (5/69) for MAA, which was dramatically higher than that for MAM (5%, *P* = 0.003). For other agents, resistance rates were not different between the two species (*P* > 0.05).

### Demographic and clinical characteristics of patients with CLA-resistant MI infections

In the same previous study demonstrated that approximately one-fifteenth of pulmonary tuberculosis (PTB) patients are afflicted with nontuberculous mycobacterial infections in China. NTM pulmonary disease patients are prone to exhibit less severe clinical symptoms than PTB patients. Females, elderly people, and patients with bronchiectasis or COPD are at high risk for developing NTM pulmonary disease [15]. So we compared the percentages of demographic and clinical characteristics of patients with CLA-susceptible and CLA-resistant MI isolates. As shown in Table 3, the majority of patients with CLA-resistant MI were male aged 45–64 years; whereas our data revealed no statistically significant difference in several demographic characteristics, including sex and age groups. In regards to the distribution of CLA-resistant MI among different clinical symptom groups, the percentage of patients presenting fever in the CLA-susceptible group (6%) was significantly higher than that in the CLA-resistant group (2%), suggesting that patients in the CLA-resistant group were likely to have milder clinical symptoms (OR [95% CI]: 0.429[0.211–0.873], *P* = 0.020). Furthermore, there were no statistical differences between two groups among comorbidities and other symptoms.

**Table 3:** Demographic and clinical characteristics associated with clarithromycin resistance in our cohort

Characteristics	No. of isolates *n* = 244 (%)	Clarithromycin		OR (95%CI)	*P* value
		Susceptible*n* = 192(79%)	Resistant *n* = 52(21%)		
Sex					
Male	124(51)	94(49)	30(58)	1.000	Reference
Female	120(49)	98(51)	22(42)	0.969(0.525~1.789)	0.920
Age, year					
<45	26(9)	20(10)	6(10)	1.000	Reference
45–65	127(42)	98(51)	29(56)	2.500(0.934~6.692)	0.068
> 65	91(48)	74(38.5)	17(33)	1.408(0.695~2.853)	0.343
Treatment history					
New cases	125(51)	96(50)	29(56)	1.000	Reference
Retreated cases	119(49)	96(50)	23(44)	1.248(0.674~2.311)	0.482
Complication					
COPD	31(13)	23(12)	8(15)	0.796(0.333~1.901)	0.607
Bronchiectasis	103(42)	79(41)	24(46)	0.895(0.482~1.662)	0.725
Asthma	6(2)	4(2)	2(4)	0.568(0.101~3.193)	0.521
Diabetes	28(11)	19(10)	9(17)	0.568(0.237~1.326)	0.188
HIV	10(4)	8(4)	2(4)	1.170(0.241~5.685)	0.846
Clinical symptoms					
Cough	82(34)	71(37)	11(21)	0.181(0.024~1.388)	0.100
Hemoptysis	4(2)	3(2)	1(2)	0.581(0.278~1.214)	0.149
Fever	12(5)	11(6)	1(2)	0.429(0.211~0.873)	0.020
Thoracalgia	7(3)	6(3)	1(2)	0.754(0.298~1.906)	0.551

OR, odds ratio; CI, confidence interval; COPD, chronic obstructive pulmonary disease

## Discussion

In the present study, we described the susceptibility profiles of the predominant NTM species from pulmonary disease patients in China. Our results demonstrated that approximate one fifth of MI isolates tested were resistant to CLA. This prevalence was dramatically higher than those in Germany (7/77, 9.1%) [17] and Sweden (15/229, 6.5%) [18] and Korea (45/818,5.5%) [19] cohort. We speculated several plausible explanations for the geographic difference in the prevalence of CLA resistance in clinical MI isolates. On the one hand, fluroquinolone resistance among *Mycobacterium tuberculosis* increased significantly in China over the past decades [20], which was regarded as a result of antibiotic abuse in China [21, 22]. It is worth mentioning that macrolides are frequently used as antibacterial agents [23]; therefore, the empirical abuse of CLA raise the possibility of accelerated development of resistance in MI isolates. On the other hand, despite limited evidence for transmission of NTM between patients, Bryant and colleagues found that the clustered NTM strains had high level, constitutive CLA resistance than uncluttered strains, indicating that the highly drug-resistant organisms are significantly more likely to cause infections in susceptible individuals [24]. Thus, this geographic diversity may reflect the predominance of CLA-resistant MI in China. Although the exact reasons remain unclear, there is growing awareness of the possibility of poor treatment outcomes in patients with MI diseases in view of the fact that CLA is the cornerstone for NTM therapy.

In addition, we found extremely high rates of resistance to multiple antimicrobial agents in clinical MI isolates, including MXF (81%) and LZD (69%). This observation appeared consistent with previous reports from 84% [18] and 59% [19], which demonstrated that these agents were ineffective against nearly half of MI isolates tested. These findings raise concerns about the clinical efficacy of guideline-endorsed therapeutic drugs used in the management of NTM pulmonary diseases [9]. Creative and focused efforts are urgently required to boost the antimycobacterial pipeline and develop novel antibiotics to fight the threat of MI infections.

Another interesting finding of the present study was that MAA was more resistant to CLA than MAM irrespective of acquired or inducible resistance, which was identical to findings reported previously [25]. Another report showed that Unlike MAA, the bacteriological response of MAM to combination antibiotic therapies containing clarithromycin was excellent [26]. Similarly, there are increasing evidence that MAA showed different drug susceptibility profiles despite sharing a very high degree of sequence identify and phenotypic pattern. In a previous study, resistance to tigecycline was more frequently observed in the MAA than in the MAM group [27]. Additionally, Cho et al. revealed that the resistance rate of MAA to MFX was remarkably higher than that of MAM [28]. Taken together, these observations suggest that MAA is likely to be more prone to accumulate antibiotic resistance than MAM, indicating that treatment of MAA disease may be more difficult.

In patients with MI infection, the clinical manifestations caused by CLA-resistant bacilli seemed to be milder than in CLA-susceptible group. There was a lower likelihood of fever in CLA-resistant MI disease in our cohort. The underlying cause of this phenomenon remains unclear but is presumably associated with loss of virulence of these drug-resistant isolates. Consistent with these observations were the findings of a murine study in which a significant number of drug-resistant tubercle bacilli tended to present low virulence [29]. It is believed that drug resistance-conferring mutations frequently lead to fitness costs in bacteria [30, 31], thereby negatively affecting the bacterial growth and survival in vivo, and showing milder clinical signs. Further experimental evidence is required to elucidate the correlation between drug resistance and virulence of NTM isolates.

We also acknowledged several limitations to the present study. First, only the predominant NTM species were included in this analysis, thus hampering determination of in vitro susceptibilities for these minority species. Second, the small sample when divided to each pilot may hamper the accurate estimation of geographic diversity and temporal trends of antimicrobial resistance in NTM. Third, although several tentative breakpoints for antimicrobial agents were provided by CLSI, the poor *in vitro-in vivo* correlations for the majority of these agents emphasize the urgent need to retrospectively analyze the therapy regimens and clinical outcomes of NTM patients; however, these data were inaccessible in our cohort. Fourth, multiple genes conferring resistance have been identified in various NTM species, which were not analyzed by using DNA sequencing approaches. Fifth, the higher proportion of CLA-resistance was noted in MAA compared with MAM. However, the molecular mechanism associated with this phenomenon remains unclear. Further experiments are warranted to elucidate the underlying mechanism.

To conclude, the study describes the susceptibility profiles of the predominant NTM species from pulmonary disease patients in China. Our data demonstrate that approximate one fifth of MI isolates tested were resistant to CLA, and extremely high rates of resistance to MFX and LZD are noted in clinical MI isolates. In addition, we have identified a higher proportion of CLA-resistant MAA isolates than MAM. The patients caused by CLA-resistant MI are at low risk for presenting with fever relative to CLA-susceptible group. More efforts are urgently required to boost the antimycobacterial pipeline and develop novel antibiotics to fight the threat of NTM infections.

## Electronic supplementary material

Below is the link to the electronic supplementary material.

Supplementary Material 1

## Data Availability

The datasets used and/or analyzed during the current study available from the corresponding author on reasonable request.
