# Supplementary material for: In vitro susceptibility of nontuberculous mycobacteria in China
Source: BMC Infect Dis. 2024 Jan 23;24:118. doi: 10.1186/s12879-024-09016-6 (PMC10804778; doi:10.1186/s12879-024-09016-6)
Supplement: Supplementary file 1 — Supplementary Material 1 [file 12879_2024_9016_MOESM1_ESM.docx]

**Supplementary Tables**

Table S1: Antimycobacterial Agents and Breakpoints for Testing MI

| Antimicrobial Agent | Breakpoints, μg/mL | | |
| --- | --- | --- | --- |
|  | Susceptible | Intermediate | Resistant |
| Clarithromycin | ≤8 | 16 | ≥32 |
| amikacin | ≤16 | 32 | ≥64 |
| moxifloxacin | ≤1 | 2 | ≥4 |
| linezolid | ≤8 | 16 | ≥32 |
| rifabutin | ≤2 | - | ≥4 |
| rifampin | ≤1 | - | ≥2 |
| ciprofloxacin | ≤1 | 2 | ≥4 |
| doxycycline | ≤1 | 2-4 | ≥8 |
| ethambutol | ≤1 | 2-4 | ≥8 |
| trimethoprim-sulfamethoxazole | ≤2/38 | - | ≥4/76 |

Abbreviations: MI, *Mycobacterium intracellulare.*

Table S2: Antimycobacterial Agents and Breakpoints for Testing RGM

| Antimicrobial Agent | Breakpoints, μg/mL | | |
| --- | --- | --- | --- |
|  | Susceptible | Intermediate | Resistant |
| Clarithromycin | ≤2 | 4 | ≥8 |
| amikacin | ≤16 | 32 | ≥64 |
| Cefoxitin | ≤16 | 32-64 | ≥128 |
| ciprofloxacin | ≤1 | 2 | ≥4 |
| doxycycline | ≤1 | 2-4 | ≥8 |
| linezolid | ≤8 | 16 | ≥32 |
| Imipenem | ≤4 | 8-16 | ≥32 |
| moxifloxacin | ≤1 | 2 | ≥4 |
| trimethoprim-sulfamethoxazole | ≤2/38 | - | ≥4/76 |
| tobramycin | ≤2 | 4 | ≥8 |

Abbreviations: RGM, rapidly-growing mycobacteria
